# Supplementary material for: Modeling glioblastoma heterogeneity as a dynamic network of cell states
Source: Mol Syst Biol. 2021 Sep 16;17(9):e10105. doi: 10.15252/msb.202010105 (PMC8444284; doi:10.15252/msb.202010105)
Supplement: Supplementary file 6 — Source Data for Figure 5 [file MSB-17-e10105-s004.zip › Figure5A_sourcedata/GSEA_3071/nosigdb_stateIII.GseaPreranked.1622795155562/IVY_CTMVP.html]

Details for gene set IVY\_CTMVP[GSEA]

|  || Dataset | state33071 |
| Phenotype | NoPhenotypeAvailable |
| Upregulated in class | na\_neg |
| GeneSet | IVY\_CTMVP |
| Enrichment Score (ES) | -0.5922671 |
| Normalized Enrichment Score (NES) | -3.241765 |
| Nominal p-value | 0.0 |
| FDR q-value | 0.0 |
| FWER p-Value | 0.0 |
Table: GSEA Results Summary

  

Fig 1: Enrichment plot: IVY\_CTMVP      
 Profile of the Running ES Score & Positions of GeneSet Members on the Rank Ordered List

  

| PROBE | GENE SYMBOL | GENE\_TITLE | RANK IN GENE LIST | RANK METRIC SCORE | RUNNING ES | CORE ENRICHMENT || 1 | MYL9 |  |  | 293 | 0.293 | -0.0671 | No |
| 2 | TAGLN |  |  | 407 | 0.257 | -0.0887 | No |
| 3 | FILIP1L |  |  | 454 | -0.252 | -0.0931 | No |
| 4 | CRIP1 |  |  | 561 | -0.258 | -0.1129 | No |
| 5 | OLFML2A |  |  | 635 | -0.261 | -0.1241 | No |
| 6 | PXDN |  |  | 666 | -0.263 | -0.1241 | No |
| 7 | LAMC1 |  |  | 1120 | -0.286 | -0.2327 | No |
| 8 | RAMP2 |  |  | 1137 | -0.286 | -0.2284 | No |
| 9 | FSTL1 |  |  | 1254 | -0.293 | -0.2497 | No |
| 10 | COL18A1 |  |  | 1302 | -0.295 | -0.2532 | No |
| 11 | ARHGAP29 |  |  | 1614 | -0.313 | -0.3243 | No |
| 12 | INPP4B |  |  | 1618 | -0.313 | -0.3158 | No |
| 13 | PDE5A |  |  | 1869 | -0.328 | -0.3707 | No |
| 14 | KCNJ2 |  |  | 1974 | -0.334 | -0.3878 | No |
| 15 | ITGA4 |  |  | 1986 | -0.334 | -0.3807 | No |
| 16 | PLAC9 |  |  | 2086 | -0.341 | -0.3963 | No |
| 17 | MFAP2 |  |  | 2169 | -0.347 | -0.4072 | No |
| 18 | TM4SF1 |  |  | 2186 | -0.348 | -0.4011 | No |
| 19 | FOXS1 |  |  | 2195 | -0.349 | -0.3929 | No |
| 20 | LAMB1 |  |  | 2556 | -0.378 | -0.4747 | No |
| 21 | ISLR |  |  | 2675 | -0.388 | -0.4938 | No |
| 22 | ACTA2 |  |  | 2721 | -0.393 | -0.4938 | No |
| 23 | SVIL |  |  | 2912 | -0.409 | -0.5308 | No |
| 24 | SERPINH1 |  |  | 2921 | -0.410 | -0.5208 | No |
| 25 | OLFML2B |  |  | 3050 | -0.425 | -0.5413 | No |
| 26 | APOLD1 |  |  | 3173 | -0.441 | -0.5598 | No |
| 27 | CD248 |  |  | 3198 | -0.442 | -0.5530 | No |
| 28 | EDNRA |  |  | 3351 | -0.471 | -0.5784 | Yes |
| 29 | MCAM |  |  | 3353 | -0.472 | -0.5647 | Yes |
| 30 | ANGPT2 |  |  | 3360 | -0.473 | -0.5523 | Yes |
| 31 | CCDC102B |  |  | 3464 | -0.497 | -0.5643 | Yes |
| 32 | PLXDC1 |  |  | 3472 | -0.499 | -0.5514 | Yes |
| 33 | OLFML3 |  |  | 3503 | -0.509 | -0.5441 | Yes |
| 34 | F2R |  |  | 3538 | -0.523 | -0.5374 | Yes |
| 35 | ITGB1 |  |  | 3574 | -0.537 | -0.5306 | Yes |
| 36 | LAMA4 |  |  | 3587 | -0.542 | -0.5178 | Yes |
| 37 | CTHRC1 |  |  | 3591 | -0.544 | -0.5025 | Yes |
| 38 | THBS1 |  |  | 3599 | -0.547 | -0.4882 | Yes |
| 39 | IGFBP7 |  |  | 3611 | -0.554 | -0.4746 | Yes |
| 40 | CFH |  |  | 3653 | -0.582 | -0.4681 | Yes |
| 41 | PRSS23 |  |  | 3658 | -0.584 | -0.4519 | Yes |
| 42 | COL4A2 |  |  | 3664 | -0.586 | -0.4359 | Yes |
| 43 | PDGFRB |  |  | 3678 | -0.598 | -0.4216 | Yes |
| 44 | DCN |  |  | 3723 | -0.633 | -0.4143 | Yes |
| 45 | COL5A2 |  |  | 3726 | -0.636 | -0.3960 | Yes |
| 46 | STC1 |  |  | 3770 | -0.702 | -0.3864 | Yes |
| 47 | PCOLCE |  |  | 3777 | -0.708 | -0.3671 | Yes |
| 48 | TGFBI |  |  | 3781 | -0.713 | -0.3468 | Yes |
| 49 | PDGFB |  |  | 3803 | -0.751 | -0.3301 | Yes |
| 50 | BGN |  |  | 3812 | -0.761 | -0.3097 | Yes |
| 51 | COL4A1 |  |  | 3829 | -0.791 | -0.2905 | Yes |
| 52 | FN1 |  |  | 3850 | -0.842 | -0.2708 | Yes |
| 53 | LUM |  |  | 3861 | -0.870 | -0.2477 | Yes |
| 54 | A2M |  |  | 3890 | -1.053 | -0.2239 | Yes |
| 55 | COL6A3 |  |  | 3900 | -1.171 | -0.1917 | Yes |
| 56 | CTGF |  |  | 3901 | -1.185 | -0.1567 | Yes |
| 57 | COL1A1 |  |  | 3902 | -1.191 | -0.1215 | Yes |
| 58 | COL1A2 |  |  | 3906 | -1.278 | -0.0846 | Yes |
| 59 | COL3A1 |  |  | 3918 | -1.418 | -0.0456 | Yes |
| 60 | MGP |  |  | 3927 | -1.633 | 0.0005 | Yes |
Table: GSEA details [plain text format]

  

Fig 2: IVY\_CTMVP: Random ES distribution      
 Gene set null distribution of ES for **IVY\_CTMVP**
